# Obesity as Assessed by Body Adiposity Index and Multivariable Cardiovascular Disease Risk

**DOI:** 10.1371/journal.pone.0094560

**Published:** 2014-04-08

**Authors:** Satvinder S. Dhaliwal, Timothy A. Welborn, Louise G. H. Goh, Peter A. Howat

**Affiliations:** 1 Curtin University, Perth, Australia; 2 Sir Charles Gairdner Hospital, Perth, Australia; University of Tolima, Colombia

## Abstract

To assess the role of body adiposity index (BAI) in predicting cardiovascular disease (CVD) and coronary heart disease (CHD) mortality, in comparison with body mass index (BMI), waist circumference (WC), and the waist circumference to hip circumference ratio (WHR). This study was a prospective 15 year mortality follow-up of 4175 Australian males, free of heart disease, diabetes and stroke. The Framingham Risk Scores (FRS) for CHD and CVD death were calculated at baseline for all subjects. Multivariable logistic regression was used to assess the effects of the measures of obesity on CVD and CHD mortality, before adjustment and after adjustment for FRS. The predictive ability of BAI, though present in the unadjusted analyses, was generally not significant after adjustment for age and FRS for both CVD and CHD mortality. BMI behaved similarly to BAI in that its predictive ability was generally not significant after adjustments. Both WC and WHR were significant predictors of CVD and CHD mortality and remained significant after adjustment for covariates. BAI appeared to be of potential interest as a measure of % body fat and of obesity, but was ineffective in predicting CVD and CHD.

## Introduction

There is a world-wide pandemic of obesity and its severe consequences affect both developed and developing countries. The body adiposity index (BAI) is proposed as a useful parameter to assess obesity [1]. BAI is simple-to-use and it does not require the assessment of body weight. Similar linear relationships between BAI and percentage (%) body fat were observed in men and women, thus suggesting that sex-specific adjustment of BAI to estimate % body fat may not be required. It was developed on the basis of showing a strong correlation with % body fat as assessed using dual-energy x-ray absorptiometry (DXA) [1]–[7]. Both hip circumference (HC) and height were strongly associated with % body fat and were included in the calculation of BAI. The formula derived was:
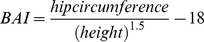



BAI is promoted as a simple technique that does not require scales and can be used in remote locations without equipment.

The body mass index (BMI (kg/m^2^)  =  weight/height^2^) remains the international standard for assessing obesity in epidemiological and clinical settings, even though there are recognised limitations influencing its validity which include sex, ethnicity, frame size and age [8]–[10]. BMI also does not differentiate between muscle mass and fat mass [11]. Despite these limitations, the American Heart Association recommends BMI to be used as a primary tool for assessing body fatness because of its global acceptance and ease of calculation.

While it is clear that controversy exists as to the best and most simple measure to use to assess adiposity [12], comparative outcome data is essential to resolve this debate. Studies have assessed the association between BAI and health risks, however, there is a need to further clarify the clinical utility of BAI in assessing body adiposity and its association with diseases [2], [3], [6], [13]–[17]. In this regard, we had the opportunity to assess the role of BAI in predicting cardiovascular disease (CVD) and coronary heart disease (CHD) mortality in comparison with BMI and measures of central obesity which includes waist circumference (WC) and the waist circumference to hip circumference ratio (WHR) in a population sample followed for 15 years.

## Methods

### Ethics statement

The Australian Institute of Health Interim Ethics Committee provided ethical clearance for the 1989 third Risk Factor Prevalence Survey, after consultation with the Commonwealth Privacy Commissioner. Participation was entirely voluntary. Participants signed an informed consent form to indicate their willingness to participate in the survey [18]. The subsequent linkage and analyses of the survey data with the National Death Index were approved by the current Ethics Committee of the Australian Institute of Health and Welfare.

### Study participants

The Australian third Risk Factor Prevalence Survey was conducted in 1989 by the National Heart Foundation (NHF) to provide a picture of the level of risk factors in the population of registered voters living in Australian capital cities [18]. The city catchment areas were Sydney (North and South), Melbourne, Brisbane, Adelaide, Perth, Hobart, Darwin and Canberra. From the initial age and sex stratified sample of 9279 Australian residents, 4175 males who were free of heart disease, diabetes or stroke at baseline were selected for this study. Respondents were mainly of European descent.

### Risk factor measurements

At baseline, fasting serum lipid levels, systolic and diastolic blood pressure, and smoking habits were recorded [19]. The Framingham Risk Scores (FRS) for CHD and CVD death were calculated at baseline for all 4175 males, using the equations from Anderson's paper [20]. The FRS were calculated from the baseline data of subjects free of heart disease, diabetes or stroke, after accounting for age, gender, systolic blood pressure, total cholesterol, high density lipoprotein cholesterol and cigarette smoking.

Physical measurements were made in light clothing and without shoes. General obesity was assessed by BAI and BMI using standardised methods [21]. BAI, which correlates well with % body fat using DXA, was calculated with the formula [1]. Central obesity was assessed by WC and WHR, using two observers by standardised methods [21]–[23]. WC was measured from the front at the narrowest point between the rib cage and the iliac crest after full expiration and HC was assessed from the side at the maximal extension of the buttocks using a metal tape, with no compression of the skin. The mean was calculated from the measurements (to the nearest centimetre) of the two observers. Height was measured to the nearest centimetre and weight to the nearest 0.1 kilogram (kg), with 1 kg deducted for light street clothing.

### Cardiovascular disease outcomes

The data on these subjects were linked with the National Death Index to determine the causes of death for 346 subjects who had died by December 31, 2004. Of the 4175 males (age 42.3±13.1 years), 88 died from CVD and 64 were from CHD. The causes of death were coded according to the 10th revision of the International Classification of Diseases (ICD-10): Codes I 00.0 to I 99.0 for CVD deaths and codes I 20.0 to I 25.9 for CHD deaths [24].

### Statistical analysis

Spearman's rank correlation was used to assess the association between the measures of general and central obesity, and CVD risk while accounting for fasting serum lipid levels, and systolic and diastolic blood pressure. The data on the general and central obesity variables were also divided into tertiles (low, medium and high tertiles, respectively) for further analysis using regression techniques. Multivariable logistic regression was used to assess the effects of the measures of obesity on CVD and CHD mortality using the 1989 NHF cohort, before adjustment (crude) and after adjustment for FRS variables (age, systolic blood pressure, total cholesterol, high density lipoprotein cholesterol and cigarette smoking). The effects of obesity variables and FRS on CVD and CHD mortality were expressed as odds-ratios and associated 95% confidence intervals. P-values less than 0.05 were considered to be statistically significant. Data was analysed using IBM SPSS Statistics Version 19.

## Results

The characteristics of the baseline cohort of 4175 males without a history of angina, diabetes or stroke are presented in Table 1. During the 15-year mortality follow-up, there were 346 deaths in total, 88 deaths were due to CVD and 64 deaths were due to CHD. Subjects who died from all-causes had higher obesity measures, serum lipid and blood pressure levels, compared to the total cohort. Those who experienced CVD and CHD deaths had higher measures of general and central obesity, compared to those who experienced non-CVD deaths. FRS was also higher in subjects who died from CVD and CHD as compared to those who experienced non-CVD deaths and the total cohort.

**Table 1:** Characteristics of cohort without angina, diabetes or stroke at baseline.

	CVD Deaths	CHD Deaths	Non-CVD Deaths	Total Cohort
Count	88	64	258	4175
Age (years)	59.4 ± 10.6	59.6 ± 10.0	56.0 ± 11.4	42.3 ± 13.1
Current smoker (%)	35.2	40.6	36.4	27.9
Weight (kg)	81.2 ± 14.0	81.2 ± 11.8	77.9 ± 12.4	79.2 ± 12.3
Height (cm)	173.6 ± 7.0	173.9 ± 7.2	173.5 ± 7.1	175.3 ± 6.9
Waist circumference (cm)	96.7 ± 10.5	96.7 ± 9.6	92.3 ± 11.4	89.9 ± 10.4
Hip circumference (cm)	102.6 ± 7.7	102.5 ± 6.2	101.1 ± 8.2	100.6 ± 7.2
Body Mass Index (kg/m^2^)	26.8 ± 4.0	26.8 ± 3.4	25.9 ± 3.7	25.8 ± 3.5
Body Adiposity Index (%)	27.0 ± 3.2	26.9 ± 2.6	26.3 ± 3.8	25.4 ± 3.4
Waist to Hip ratio	0.94 ± 0.06	0.94 ± 0.06	0.91 ± 0.07	0.89 ± 0.06
Total cholesterol (mmol/L)	5.9 ± 1.0	5.9 ± 1.0	5.8 ± 1.1	5.5 ± 1.1
HDL cholesterol (mmol/L)	1.1 ± 0.3	1.1 ± 0.3	1.3 ± 0.4	1.2 ± 0.3
Total cholesterol to HDL ratio	5.5 ± 1.7	5.7 ± 1.8	5.0 ± 1.7	4.9 ± 1.6
Systolic Blood Pressure (mm Hg)	140.6 ± 18.4	141.4 ± 18.8	140.6 ± 21.3	128.5 ± 16.2
Diastolic Blood Pressure (mm Hg)	86.2 ± 11.6	86.0 ± 11.8	84.8 ± 11.5	81.0 ± 10.9
Framingham predicted risk - CVD death (%)	17.0 ± 11.2	-	13.3 ± 10.0	5.1 ± 7.0
Framingham predicted risk - CHD death (%)	-	13.0 ± 8.0	9.7 ± 7.2	3.7 ± 5.5

Abbreviations: CVD, cardiovascular disease; CHD, coronary heart disease; HDL cholesterol, high density lipoprotein cholesterol.

Table 2 presents the distribution of CVD deaths, CHD deaths and non-CVD deaths across the tertiles of obesity related measures, where low, medium and high represents the 1^st^, 2^nd^ and 3^rd^ tertile, respectively. A trend towards a greater proportion of deaths in the higher tertiles was evident in all obesity related measures. There was, however, a steeper trend in WHR and WC compared to BAI, BMI and HC, indicating a stronger association for these measures between tertiles and deaths due to CVD and CHD.

**Table 2:** Distribution* of cardiovascular disease (CVD) deaths and coronary heart disease (CHD) deaths by tertiles of obesity related measures.

		CVD Deaths	CHD Deaths	Non-CVD Deaths
Count		88	64	258
Body Adiposity Index (BAI)	Low (%)	16.5	11.5	29.2
	Medium (%)	32.9	37.7	27.2
	High (%)	50.6	50.8	43.6
Body Mass Index (BMI) (kg/m^2^)	Low (%)	22.6	16.7	32.9
	Medium (%)	28.6	31.7	29.8
	High (%)	48.8	51.7	37.3
Waist Circumference (WC) (cm)	Low (%)	8.0	7.9	26.0
	Medium (%)	33.3	31.7	30.2
	High (%)	58.6	60.3	43.8
Hip Circumference (HC) (cm)	Low (%)	23.0	17.5	34.5
	Medium (%)	31.0	31.7	29.8
	High (%)	46.0	50.8	35.7
Waist to Hip ratio (WHR)	Low (%)	6.9	6.3	22.1
	Medium (%)	26.4	25.4	32.9
	High (%)	66.7	68.3	45.0

* L°w, medium and high refers to 1^st^, 2^nd^ and 3^rd^ tertiles, respectively.

Abbreviations: CVD, cardiovascular disease; CHD, coronary heart disease.

The associations between obesity related measures are presented in Table 3. All correlations were significant (*p*<0.001) but caution should be exercised in the interpretation of high correlations, as computed variables may use other obesity related measures in its calculations. The Spearman's rank correlation between BAI and BMI, WC and HC was higher compared to the correlation with WHR. BMI was more highly correlated with BAI, WC and HC compared to the correlation with WHR.

**Table 3:** Spearman's rank correlation between obesity related measures.

**Weight (kg)**	1						
**Height (cm)**	0.449	1					
**BMI (kg/m^2^)**	0.833	−0.068	1				
**Waist (cm)**	0.800	0.109	0.842	1			
**Hip (cm)**	0.866	0.294	0.794	0.807	1		
**WHR**	0.476	−0.092	0.603	0.830	0.373	1	
**BAI**	0.398	−0.500	0.766	0.629	0.632	0.415	1
	**Weight**	**Height**	**BMI**	**Waist**	**Hip**	**WHR**	**BAI**

All correlations were significant (p-values < 0.001). Exercise caution in the interpretation of correlation coefficients as computed variables may utilise other variables in its calculation. Abbreviations: BMI, body mass index; waist, waist circumference; hip, hip circumference; WHR, waist to hip ratio; BAI, body adiposity index.

Table 4 presents the Spearman's rank correlation between obesity related measures and the continuous variables used in the calculation of the FRS. Although all correlations were statistically significant (*p*<0.001), BAI and BMI did not appear to correlate as highly with the FRS variables as WC and WHR.

**Table 4:** Spearman's rank correlation between measures of obesity and Framingham predictor variables.

	BMI	Waist	WHR	BAI
**Systolic Blood Pressure**	0.317	0.356	0.323	0.280
**Diastolic Blood Pressure**	0.361	0.391	0.342	0.297
**Total Cholesterol**	0.265	0.310	0.319	0.254
**HDL Cholesterol**	−0.256	−0.249	−0.221	−0.171
**Triglycerides**	0.390	0.433	0.427	0.315
**Total Cholesterol to HDL ratio**	0.383	0.404	0.386	0.301

All correlations were significant (p-values <0.001).

Abbreviations: BMI, body mass index; waist, waist circumference; WHR, waist to hip ratio; BAI, body adiposity index; HDL Cholesterol, high density lipoprotein cholesterol.

The results of the multivariable logistic regression used to assess the effects of measures of obesity on CVD and CHD deaths are presented in Table 5 and Table 6, respectively. The effects of BMI, WC, HC, WHR and BAI on CVD and CHD mortality were first presented without adjustment (crude effects), then adjusted for by age and finally adjusted for FRS variables (age, systolic blood pressure, total cholesterol, high density lipoprotein cholesterol and cigarette smoking). The predictive ability of BAI, though present in the unadjusted analyses, was generally not significant after adjustment for age and FRS for both CVD and CHD mortality. BMI behaved similarly to BAI in that its predictive ability was generally not significant after adjustments. Both WC and WHR were significant predictors of CVD and CHD mortality and remained significant after adjustment for covariates. The odds-ratios for the comparison between the highest tertile and the lowest tertile for CVD mortality was 3.84 (1.59–9.25) for WC and 5.42 (2.12–13.89) for WHR. In relation to CHD mortality, the odds-ratios for the comparison between the highest tertile and the lowest tertile was 3.16 (1.19–8.37) for WC and 4.47 (1.55–12.89) for WHR.

**Table 5:** Odds-ratios and associated 95% confidence intervals for measures of obesity at baseline for cardiovascular disease (CVD) mortality using multivariable logistic regression.

	Unadjusted	Adjusted by age	Adjusted by FRS for CVD mortality
***Body Adiposity Index (BAI)***			
FRS for CVD Mortality			3.02 (2.50–3.64) *(p < 0.001)*
Age		1.13 (1.10–1.16) *(p < 0.001)*	
BAI tertiles:			
Medium vs. Low	2.02 (1.06–3.85) *(p = 0.033)*	1.34 (0.69–2.61) *(p = 0.381)*	1.42 (0.72–2.82) *(p = 0.316)*
High vs. Low	3.14 (1.71–5.76) *(p < 0.001)*	1.32 (0.70–2.47) *(p = 0.393)*	1.50 (0.77–2.91) *(p = 0.233)*
***Body Mass Index (BMI)***			
FRS for CVD mortality			3.08 (2.56–3.70) *(p < 0.001)*
Age		1.13 (1.10–1.16) *(p < 0.001)*	
BMI tertiles:			
Medium vs. Low	1.27 (0.69–2.33) *(p = 0.443)*	0.99 (0.53–1.85) *(p = 0.979)*	1.07 (0.57–2.03) *(p = 0.829)*
High vs. Low	2.19 (1.26–3.79) *(p = 0.005)*	1.47 (0.83–2.58) *(p = 0.184)*	1.35 (0.74–2.44) *(p = 0.326)*
***Waist Circumference (WC)***			
FRS for CVD Mortality			2.83 (2.34–3.41) *(p < 0.001)*
Age		1.12 (1.10–1.15) *(p < 0.001)*	
WC tertiles:			
Medium vs. Low	4.38 (1.91–10.03) *(p < 0.001)*	2.46 (1.06–5.71) *(p = 0.036)*	3.28 (1.34–8.03) *(p = 0.009)*
High vs. Low	7.77 (3.51–17.18) *(p < 0.001)*	3.15 (1.40–7.07) *(p = 0.005)*	3.84 (1.59–9.25) *(p = 0.003)*
***Hip Circumference (HC)***			
FRS for CVD Mortality			3.06 (2.56–3.67) *(p < 0.001)*
Age		1.13 (1.10–1.15) *(p < 0.001)*	
HC tertiles:			
Medium vs. Low	1.36 (0.76–2.43) *(p = 0.303)*	1.05 (0.58–1.91) *(p = 0.872)*	1.19 (0.64–2.21) *(p = 0.575)*
High vs. Low	2.15 (1.25–3.70) *(p = 0.006)*	1.40 (0.80–2.45) *(p = 0.235)*	1.44 (0.80–2.59) *(p = 0.219)*
***Waist to Hip ratio (WHR)***			
FRS for CVD Mortality			2.75 (2.28–3.33) *(p < 0.001)*
Age		1.12 (1.09–1.15) *(p < 0.001)*	
WHR tertiles:			
Medium vs. Low	3.89 (1.58–9.57) *(p = 0.003)*	2.30 (0.92–5.73) *(p = 0.074)*	3.11 (1.16–8.33) *(p = 0.024)*
High vs. Low	10.06 (4.33–23.39) *(p < 0.001)*	3.82 (1.62–9.01) *(p = 0.002)*	5.42 (2.12–13.89) *(p < 0.001)*

Abbreviations: FRS, Framingham Risk Scores; CVD, cardiovascular disease.

**Table 6:** Odds-ratios and associated 95% confidence intervals for measures of central obesity at baseline for coronary heart disease (CHD) mortality using multivariable logistic regression.

	Unadjusted	Adjusted by age	Adjusted by FRS for CHD mortality
***Body Adiposity Index (BAI)***			
FRS for CHD Mortality			4.28 (3.21–5.69) *(p < 0.001)*
Age		1.13 (1.10–1.16) *(p < 0.001)*	
BAI tertiles:			
Medium vs. Low	3.32 (1.42–7.76) *(p = 0.006)*	2.23 (0.94–5.29) *(p = 0.068)*	2.17 (0.91–5.17) *(p = 0.081)*
High vs. Low	4.50 (1.98–10.26) *(p < 0.001)*	1.89 (0.82–4.39) *(p = 0.137)*	2.04 (0.87–4.80) *(p = 0.103)*
***Body Mass Index (BMI)***			
FRS for CHD mortality			4.42 (3.33–5.87) *(p < 0.001)*
Age		1.13 (1.10–1.17) *(p < 0.001)*	
BMI tertiles:			
Medium vs. Low	1.91 (0.89–4.13) *(p < 0.001)*	1.52 (0.69–3.32) *(p = 0.295)*	1.55 (0.71–3.41) *(p = 0.275)*
High vs. Low	3.14 (1.54–6.44) *(p = 0.002)*	2.12 (1.02–4.40) *(p = 0.043)*	1.81 (0.86–3.83) *(p = 0.118)*
***Waist Circumference (WC)***			
FRS for CHD Mortality			4.07 (3.05–5.43) *(p < 0.001)*
Age		1.12 (1.09–1.16) *(p < 0.001)*	
WC tertiles:			
Medium vs. Low	4.21 (1.57–11.24) *(p = 0.004)*	2.34 (0.86–6.32) *(p = 0.094)*	2.57 (0.94–6.98) *(p = 0.065)*
High vs. Low	8.04 (3.15–20.48) *(p < 0.001)*	3.22 (1.24–8.33) *(p = 0.016)*	3.16 (1.19–8.37) *(p = 0.021)*
***Hip Circumference (HC)***			
FRS for CHD Mortality			4.42 (3.35–5.83) *(p < 0.001)*
Age		1.13 (1.10–1.16) *(p < 0.001)*	
HC tertiles:			
Medium vs. Low	1.83 (0.87–3.84) *(p = 0.108)*	1.43 (0.68–3.03) *(p = 0.350)*	1.51 (0.71–3.22) *(p = 0.289)*
High vs. Low	3.13 (1.57–6.24) *(p = 0.001)*	2.06 (1.02–4.15) *(p = 0.044)*	2.00 (0.98–4.09) *(p = 0.057)*
***Waist to Hip ratio (WHR)***			
FRS for CHD Mortality			3.93 (2.93–5.25) *(p < 0.001)*
Age		1.12 (1.09–1.15) *(p < 0.001)*	
WHR tertiles:			
Medium vs. Low	4.04 (1.35–12.12) *(p = 0.013)*	2.37 (0.78–7.19) *(p = 0.126)*	2.62 (0.86–7.97) *(p = 0.09)*
High vs. Low	11.08 (3.97–30.94) *(p < 0.001)*	4.16 (1.47–11.78) *(p = 0.007)*	4.47 (1.55–12.89) *(p = 0.006)*

Abbreviations: FRS, Framingham Risk Scores; CHD, coronary heart disease.

## Discussion

### Key findings

This study showed that BAI, like BMI, predicted CVD and CHD mortality when unadjusted for age and cardiovascular risk factors. But after adjustment for FRS variables (age, systolic blood pressure, total cholesterol, high density lipoprotein cholesterol and cigarette smoking), the association became non-significant. It is of interest that BAI and BMI performed similarly. In this context, the American Heart Association advises that BMI should be used with another measure such as WC, as primary tools for assessing adiposity. Individuals with elevated BMI or a proportionally high WC for a given BMI should have other cardio-metabolic risk factors evaluated for risk stratification. BAI requires a mathematical calculation of some complexity, and this cannot be used conveniently in field studies. For all of these reasons, BAI cannot be justified as a measure of any utility [25].

In contrast, this study showed that measures of central obesity were clearly superior. Both WC and WHR predicted CVD and CHD mortality, but WHR showed the strongest risk ratios and was independent of FRS. WHR is preferred because it is free of ethnic bias whereas WC requires ethnic specific criteria [23], [26]. Both WC and WHR are very simple techniques that can be employed in remote locations without scales. We emphasise that the measurement techniques, although simple, require careful standardisation and quality control in terms of the sites of measurement. WC should be measured at the narrowest level between the ribs and hips after exhaling when viewed from the front. HC should be measured from the point of maximum buttock extent when viewed from the side. Two consecutive placements would be recorded for each site and to the nearest 1 centimetre using a non-stretchable tape on a horizontal plane without compression of the skin and the average value used. When extreme obesity exists with abdominal apron, the HC can be measured in supine position.

### Comparison with other studies

Similar results were reported in other studies [10], [11], [27]–[29]. BMI, WC and weight were more consistently correlated with cardiometabolic disease risk factors, compared to BAI, in a cohort of 698 Mexican Americans [11]. Where significant correlations were observed, BAI reported similar or weaker correlations with cardiometabolic trait, compared to BMI [11]. A cross-sectional study on Spanish Caucasian adult workers found that BAI was less correlated with CVD and metabolic risk factors, compared to BMI, WC and waist-to-height ratio (WHtR) [27]. WHtR reported the best correlations [27]. In addition, BAI had lower discriminatory capacity in diagnosing metabolic syndrome, compared to BMI [27]. BAI was also less associated with CHD risk factors, compared to BMI and WC, in another study among adults in the 1988–1994 National Health and Nutrition Examination Survey (NHANES III) [29]. It was observed that BAI provided little additional information on risk factor levels above those provided by BMI [29]. Another cross-sectional study evaluating the predictive ability of BAI, and % body fat and CVD risk factors in a Chinese population found that BAI was not a better indicator of % body fat, hypertension, dyslipidaemia, metabolic syndrome and intima-media thickening of the common carotid arteries, compared to BMI and WC [28]. These results were consistent with another study which found that BAI was inferior to BMI in predicting lipids, blood pressure and other CVD risk factors [16]. In summary, BAI does not overcome the limitations of BMI and is possibly a poorer indicator of CVD risk than BMI [10], [15], [27], [29]. Central obesity measures are better indicators of CVD risk [12], [15].

Support for WHR as the superior index of obesity for cardiovascular risk assessment is seen in large cross-sectional studies including the INTERHEART study [30] and the Obesity in Asia collaboration study [23], [31], [32]. Similarly the Dallas Heart Study demonstrated that WHR clearly outperforms WC and BMI in estimating coronary calcium scores, although the cross-sectional analysis could not identify WHR as an independent predictor compared to conventional risk factors.

### Strengths and limitations

This study provided evidence that BAI is inadequate for assessing body adiposity and its association with CVD. Measures of central obesity are better predictors of CVD compared to BAI and BMI, in men. In addition, this study was carried out using a representative sample of the Australian male population. Although there is only one set of baseline measurements recorded for some risk variables but variables including measures of obesity were measured twice.

## Conclusions

To conclude, BAI looked to be of potential interest and can be used as a measure of % body fat and of obesity, but failed to show any predictive ability for CVD and CHD deaths after age and risk factor adjustments. Conversely, measures that include an assessment of central obesity, particularly the WHR, shows strong association with cardiovascular outcomes and this measure is also quite simple to obtain in field studies and remote locations.
